# The Landscape of BRCA1‐Associated Post‐Translational Modifications

**DOI:** 10.1002/cbin.70186

**Published:** 2026-07-11

**Authors:** Nethma Hewa Waduge, Junhua Xiao, Gregory M. Davis

**Affiliations:** ^1^ Department of Biomedical, Health and Exercise Sciences Swinburne Institute of Technology Melbourne Victoria Australia

**Keywords:** acetylation, BRCA1, methylation, PARylation, phosphorylation, post‐translational modifications, PTMs, ubiquitination

## Abstract

The BRCA1‐BARD1 complex is a vital protein that promotes homologous recombination, acting through its E3 ubiquitin ligase activity. Although ubiquitination remains its only confirmed enzymatic function, emerging evidence suggests that the complex influences a broad range of post‐translational modifications (PTMs), including methylation, phosphorylation, acetylation, and PARylation, involving other proteins. This review examines findings from various eukaryotic biological models to elucidate how BRCA1 regulates key PTMs. We explore how BRCA1 shapes chromatin architecture by modulating key PTMs such as ubiquitination, methylation, and acetylation. While BRCA1‐driven changes in methylation appear to have widespread effects throughout the cell, its influence on ubiquitination and acetylation tends to be more region‐ or site‐specific. Furthermore, this review discusses BRCA1's role in modifying specific target proteins, such as PLK‐1, Aurora A, and p53, and explores its involvement in non‐ubiquitin PTMs. Beyond its well‐established role in DNA repair, BRCA1 also contributes to other cellular processes involving non‐ubiquitin PTMs, such as in meiotic sex chromosome inactivation, which is also detailed in this review. Collectively, this review expands the functional landscape of BRCA1 beyond DNA repair, providing new insights into its regulatory impact across various post‐translational pathways.

**Abbreviations**
53BP1: Tumour suppressor p53‐binding protein 1
ATM: Ataxia‐telangiectasia mutated
AURKA: Aurora kinase A
AURKB: Aurora kinase B
BARD1: BRCA1‐associated RING domain protein 1
BRCA1: Breast cancer gene 1
BRCA2: Breast cancer gene 2
brc‐1: BRCA homolog 1
brd‐1: BARD homolog 1
CHK1: Checkpoint kinase 1
CHK2: Checkpoint kinase 2
CtIP: Carboxy‐terminal binding protein
DNA: Deoxyribonucleic acid
DNMT: Deoxyribonucleic acid methyltransferase
DNMT3A: Deoxyribonucleic acid methyltransferase 3 alpha
DNMT3B: Deoxyribonucleic acid methyltransferase 3 beta
DSBs: Double‐stranded breaks
E2F1: Early 2 factor transcription factor 1
ER: Endoplasmic reticulum
ERAD: Endoplasmic reticulum associated protein degradation
ER⍺: Estrogen receptor alpha
FANCB: Fanconi anaemia group B protein
H2A: Histone 2 A
H3: Histone 3
H3K9me2: di‐methylation of lysine at residue 9 on histone 3
H4: Histone 4
HP1: Heterochromatin protein 1
HR: Homologous recombination
IRE1: Inositol‐requiring enzyme 1
L1 LINEs: Long interspersed nuclear element‐1
MET‐2: Histone methyltransferase‐like 2
miR‐155: Micro ribonucleic acid ‐ 155
miR‐29: Micro ribonucleic acid ‐ 29
miR‐29b‐1‐5p: Micro RNA derived from the 5p arm of miR‐29
mRNA: Messenger ribonucleic acid
MSCI: Meiotic sex chromosome inactivation
NAD+: Oxidised nicotinamide adenine dinucleotide
NBS1: Nibrin
NHEJ: Non‐homologous end joining
p21waf1: Wild‐type p53‐activated fragment 1
PARP1: Poly [ADP‐ribose] polymerase 1
PARylation: Poly ADP‐ribosylation
PCNA: Proliferating cell nuclear antigen
PERK1: Proline‐rich receptor‐like protein kinase 1
PLK‐1: Polo‐like kinase 1
PP2Cδ: Protein phosphatase 2 C delta
PP4C: Protein phosphatase 4 catalytic subunit
PTMs: Post‐translational modifications
RIF1: Replication timing regulatory factor 1
RNAi: Ribonucleic acid interference
SKP2: S‐phase kinase‐associated protein 2
SMARCAD1: SNF2‐related chromatin remodelling ATPase with DexD box 1
ssDNA: Single stranded deoxyribonucleic acid
SUMOylation: Attachment of a small ubiquitin‐like modifier protein
UPR: Unfolded protein response
VDR: Vitamin D receptor
VDREs: Vitamin D response element

## 1 Introduction

Eukaryotic cells undergo between 10 and 50 double‐stranded breaks (DSBs) per day (Vilenchik and Knudson 2000). If unrepaired, these breaks can result in numerous diseased states, including cancer. Depending on the type of lesion, the DNA repair process varies. Alterations to nucleotide bases due to exposure to mutagens require a specialised pathway known as base excision repair (Lindahl 1974). The mismatch repair pathway repairs errors in DNA replication, and lesions that distort DNA structure are resolved by nucleotide excision repair (Kitsera et al. 2019). Because DNA repair mechanisms are complex pathways, disruption at any point can result in less faithful protection of the genome. DSBs can be repaired through less robust mechanisms, such as Non‐Homologous End Joining (NHEJ), which may lead to tumour formation and cancer (Brady et al. 2003; Singh et al. 2021). Homologous Recombination (HR) is the most accurate DNA repair mechanism, in which a homologous chromosome serves as a template (Smith et al. 2007). HR is driven by a variety of proteins involved in the DNA damage response, with the BRCA1‐BARD1 complex serving as a primary regulator and mediator (Scully et al. 1997; Westermark et al. 2003). While BRCA1 is not essential for HR, it is crucial for maintaining efficiency and preventing failures in the repair process (Reid et al. 2008). The BRCA1‐BARD1 complex possesses E3 Ubiquitin Ligase enzymatic activity (Reid et al. 2008), which is necessary for the ubiquitination of downstream targets, resulting in various fates such as proteasomal degradation, stabilisation, or activation; the outcome being dependent on the substrate and the E2 conjugating enzyme (Christensen et al. 2007). Despite this, many questions regarding ubiquitination and DNA repair remain unanswered.

BRCA1‐BARD1 takes part in many cell functions beyond HR, including cell cycle and transcriptional control (Welcsh et al. 2002; Simhadri et al. 2019). To aid this, BRCA1 influences various post‐translational modifications (PTMs) extending beyond its well‐characterised role in ubiquitination. However, the mechanisms responsible for these broad PTM‐related functions are poorly understood, partly because the only enzymatic activity that BRCA1 exhibits is that of a ubiquitin ligase (Mallery et al. 2002). Studies have shown that BRCA1 mutants lacking E3 activity retain key BRCA1 functions in DNA repair (Reid et al. 2008), raising the question of whether these functions are mediated by compensatory mechanisms or by the direct action of BRCA1. The mechanism of BRCA1's E3 ligase activity has been explored in depth, yet much of BRCA1's influence on other PTMs has largely been overlooked.

**Figure 1:**
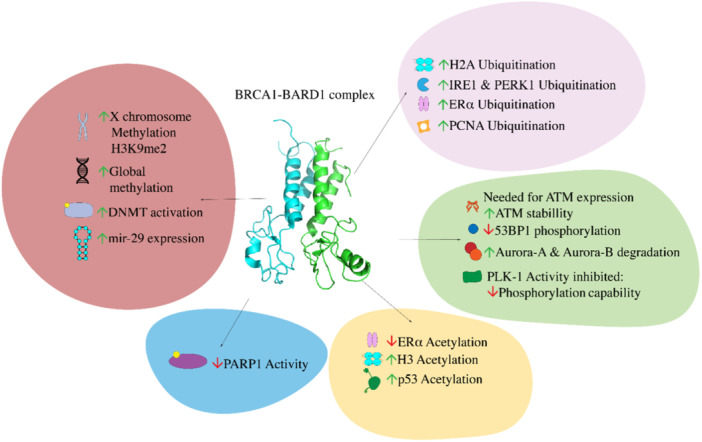
Post‐translational modifications facilitated by the BRCA1‐BARD1 complex: Currently known post‐translational modifications that BRCA1‐BARD1 influences (clockwise from right). Ubiquitination, Phosphorylation, Acetylation, PARylation, and Methylation, and the modulation of enzyme activity as reported. BRCA1‐BARD1 structural complex adapted from Brzovic et al. (2001).

Although BRCA1 is characterised by its role in ubiquitination, other PTMs have complex relationships with BRCA1. For example, BRCA1‐BARD1 is required for the expression and stability of the ATM kinase (Moiola et al. 2012; Qi et al. 2022), although BRCA1 also negatively influences the kinase activity of proteins such as PLK‐1 during genotoxic stress (Zou et al. 2013). Additionally, BRCA1 reduces acetylation of ER⍺ to decrease its activity (Ma et al. 2010), while conversely leading to an increase in acetylation of histones (Pickholtz et al. 2014) and p53 (Li et al. 2019). This results in chromatin decondensation and enhanced protein stability. BRCA1 is involved in increasing global methylation through interactions with DNMT and repressing gene expression by upregulating miR‐29, resulting in the degradation of DNMT mRNA, leading to decreased protein presence (Santarosa et al. 2023). Together, these examples highlight BRCA1 as a versatile regulator of PTMs, exerting context‐dependent effects that extend well beyond its canonical role in ubiquitination. Due to the increasing number of PTMs that BRCA1 participates in, this review will highlight the landscape of BRCA1‐BARD1‐associated PTMs reported in mammalian and other eukaryotic models. Figure 1 illustrates and summarises the post‐translational modifications addressed in this review, indicating whether the modification is applied to or removed from the target.

## 2 Recently Characterised BRCA1 Ubiquitin Targets

Many BRCA1 functions are achieved through the ubiquitination of various proteins, causing degradation, modulation of activity, and alteration of localisation. Ubiquitination occurs through the attachment of a 76‐amino acid protein known as ubiquitin (Goldstein et al. 1975). This process requires 3 enzymes; known as E1, E2, and E3 (Swatek and Komander 2016). The addition of single ubiquitin molecules can form a chain, resulting in polyubiquitination. Although the decoding of these chains is not fully understood, studies have shown that chain length and the types of linkages by which ubiquitin molecules are covalently bonded influence the processing of the molecule to which the chain is attached (Zhang et al. 2017). BRCA1 is an E3 ligase and is responsible for the addition of ubiquitin molecules from an E2 enzyme onto a substrate. In this review, only the most consequential and newly identified ubiquitination interactions will be explored (for previously identified interactions, see Witus et al. (2021)).

BRCA1‐BARD1 causes the ubiquitination of histone H2A to allow HR in response to DNA damage (Witus et al. 2021). Investigations over the last decade have shown that BRCA1‐driven ubiquitination of H2A is required for the recruitment of DNA repair proteins and for efficient DNA end resection, which are necessary for HR (Uckelmann et al. 2018). BRCA1 also promotes HR by suppressing 53BP1. While the actual mechanism of this interaction is not fully understood, Densham et al. (2016) suggest that BRCA1 causes the repositioning of 53BP1, which bars end‐resection and acts as a driver of NHEJ via ubiquitination. Furthermore, this study also suggests that ubiquitination of H2A enables SMARCAD1 recruitment, allowing 53BP1 to be repositioned and that blocks to HR are removed through chromatin remodelling, allowing efficient DNA repair.

Loss of BRCA1 promotes genomic instability and consequently leads to an increase in misfolded proteins (Konishi et al. 2011). Theoretically, an excessive number of misfolded proteins should cause cell death, a fate which a BRCA1‐deficient cell avoids (Kim et al. 2010). This was recently explained by Hromas et al. (2022), who demonstrated that in healthy cells, BRCA1 mediates the Unfolded Protein Response (UPR) via proteasomal degradation of the pathway sensors, PERK1 and IRE1. This prevents UPR activation. UPR upregulates ER chaperone proteins (Bertolotti et al. 2000) and subdues protein translation. BRCA1 causes degradation of the Endoplasmic Reticulum Stress Detectors via the Endoplasmic Reticulum‐Associated Protein Degradation (ERAD) pathway (Hromas et al. 2022), thereby suppressing UPR. Activation of UPR can lead to either the suppression or activation of apoptosis (Robinson 2006). Although the exact mechanism of this pathway remains unexplored, the availability of a survival pathway through UPR might explain why BRCA1‐deficient or dysfunctional cells prevent cell death (reviewed in Avril et al. 2017). Excessive UPR has been associated with pathological conditions, such as diabetes (Sharma et al. 2021), and has repercussions such as the shutdown of translation through PERK1 reaction pathways (Radford et al. 2015). BRCA1, which mediates proteasomal degradation of PERK1 and IRE1 via ubiquitination, likely regulates the UPR but may also play a role in maintaining the survivability of cancer cells.

In the presence of intracellular oestrogen, the oestrogen receptor (ER⍺) binds with oestrogen and causes downstream signalling. In response to this, BRCA1 ubiquitinates ER⍺ for degradation to regulate cell division and proliferation (Eakin et al. 2007; Dizin and Irminger‐Finger 2010). Interestingly, the BRCA1‐BARD1 complex is in a negative feedback loop with ER⍺, where BRCA1 causes the ubiquitin‐dependent proteasomal degradation of the receptor, and the receptor is needed for the expression of BRCA1‐BARD1. Additionally, loss of function of the RING domain of BARD1 stabilises ERα, indicating that ubiquitin activity is crucial for ERα degradation (Dizin and Irminger‐Finger 2010).

The configuration of ubiquitin chains can have multiple consequences for the attached protein. As such, BRCA1‐based ubiquitination can lead to outcomes other than degradation. For example, BRCA1 initiates non‐degradative ubiquitination of the DNA replication co‐factor, Proliferating Cell Nuclear Antigen (PCNA), allowing the enzyme to replicate longer stretches of DNA without dissociating from the strand (Boehm et al. 2016). Ubiquitination of PCNA by BRCA1 increases PCNA efficiency, as evidenced by the absence of BRCA1. This occurs through two processes, where the absence of BRCA1 E3 ligase activity results in increased replication fork collapse, as well as DNA synthesis failures in ssDNA gaps (Salas‐Lloret et al. 2024). Additionally, BRCA1‐BARD1 have also been shown to be responsible for the efficient activation of PCNA and other DNA repair proteins through ubiquitination (Tian et al. 2013).

## 3 BRCA1 Promotes Phosphorylation of DNA Repair Proteins

Primarily occurring on Serine, Threonine, and Tyrosine residues, phosphorylation is a PTM that is generally applied to control the activity of a protein (Dohadwala et al. 1994). Phosphate groups carry a strong negative charge, and the addition of this group can alter protein conformation, transitioning it from an inactive state to an active state and vice versa (Dohadwala et al. 1994). While phosphorylation is not a reaction pathway in which BRCA1 has shown enzymatic activity, BRCA1 does influence the phosphorylation of specific proteins. There are instances where this control of phosphorylation is specific; however, BRCA1 may impact nearly all DNA damage‐related phosphorylation through transcriptional regulation of ATM.

Investigations in prostate cancer cell lines have shown that BRCA1, the DNA damage repair protein, CtIP, and the transcription factor, E2F1, bind to the ATM promoter (Moiola et al. 2012). Disruption of BRCA1 or CtIP binding can lead to transcriptional repression of ATM, a protein kinase responsible for phosphorylating many DNA damage proteins, including TP53, BRCA1, CHK1, and CHK2 (Phan and Rezaeian 2021). ATM also detects DNA damage, making it one of the most important regulators of the genotoxic stress response (Canman et al. 1998). In addition, overexpression of BRCA1 results in increased ATM transcription, thereby indirectly affecting the phosphorylation of other proteins (Moiola et al. 2012). In support of the model that BRCA1 supports ATM function, BRCA1 is essential for maintaining ATM activity in mouse models, where it is involved in establishing a feedback loop that regulates ATM (Qi et al. 2022). In this case, mutations to the ATM phosphorylation site of BRCA1 resulted in a reduction of ATM autophosphorylation and phosphorylation of ATM substrates, resulting in reduced recruitment of ATM at DSB sites (Moiola et al. 2012). Reduction of ATM decreases DNA repair and reduced phosphorylation capacity may lead to genomic instability as seen in kinase‐dead ATM mutants in mice (Yamamoto et al. 2012). As ATM is a major DNA damage sensor, it is important for HR activation, NHEJ inhibition, cell cycle arrest, and management of replicative stress (Olcina et al. 2013; Bakr et al. 2015; Britton et al. 2020; Phan and Rezaeian 2021). Due to this, BRCA1 enhances ATM activity, thereby enabling greater prevention of tumour development and maintaining genomic integrity. Additionally, BRCA1 phosphorylation is necessary for the recruitment of SKP2, an E3 ubiquitin ligase which is required for the ubiquitination of the DNA damage repair protein, NBS1 and is essential for the stable activation of ATM (You et al. 2005; Kim et al. 2017; Warren and Pavletich 2022). BRCA1 promotes HR but also impedes NHEJ through facilitating the dephosphorylation of 53BP1. This interaction is dependent on PP4C, a serine/threonine phosphatase and dephosphorylation of 53BP1 leads to the release of RIF1, another driver of NHEJ from the site of DNA damage (Chapman et al. 2013; Isono et al. 2017). While the interaction between BRCA1 and PP4C is not well understood, this suggests that BRCA1 might facilitate the recruitment of regulatory subunits and other factors required for PP4C, as the loss of BRCA1 does not abolish the interaction between PP4C and 53BP1. Collectively, this suggests that phosphorylation driven by BRCA1 is necessary for efficient DNA repair, and favours HR over NHEJ.

Aurora A and B (AURKA and AURKB) kinases are cell kinases required in the cell cycle process (Liu et al. 2004; Bruinsma et al. 2014; Hadders et al. 2020). AURKA is required for centrosome development and spindle formation and phosphorylates key DNA repair proteins such as PLK1 and p53 (Liu et al. 2004; Bruinsma et al. 2014). AURKA is also required for the effective transition to mitosis from the G_2_ phase (Marumoto et al. 2002). Additionally, AURKB is required for accurate chromosomal segregation (Hadders et al. 2020). Both AURKA and AURKB are highly expressed in tumours, and mutations in either gene can cause chromosomal aneuploidy (Lu et al. 2008; González‐Loyola et al. 2015). Studies show that BRCA1/2 and Aurora A/B negatively regulate each other through proteasomal degradation (Wang et al. 2014). This was observed in ovarian cancer cells, where AURKA expression increased in response to RNAi of BRCA1 and BRCA2, as well as observations in pancreatic cells that lack BRCA2, where RNAi of AURKA and AURKB enhanced expression of BRCA1. In this instance, introduction of a 26S‐proteosome inhibitor abolishes this association, indicating that this negative regulation was due to proteasomal degradation (Wang et al. 2014). This suggests that Aurora A/B and BRCA1/2 negatively regulate each other through post‐translational modifications and that BRCA1 downregulates AURKA and AURKB, thereby affecting AURKA's and AURKB's phosphorylation targets. As these proteins are involved in cell cycle checkpoint control, it is likely that dysregulation of the balance between BRCA1/2 and Aurora A/B leads to changes to the phosphorylation landscape, impeding accurate cell cycle progression.

BRCA1 is known to regulate Polo‐Like Kinase 1 (PLK‐1), which interacts with AURKA. PLK‐1 has functions in cell cycle control, chromosome segregation, and spindle assembly (Liu and Erikson 2003; von Schubert et al. 2015; Zhang et al. 2017). Recent findings show that PLK‐1 and BRCA1 regulated each other; in cell lines, PLK‐1 increases the recruitment of BRCA1 at DSB sites, and BRCA1 decreases the phosphorylation capacity of PLK‐1 under genotoxic stress (Zou et al. 2013; Chabalier‐Taste et al. 2016). In the absence of BRCA1, PLK‐1 inhibition is reduced; however, this function is recovered by the presence of other DNA damage proteins. These attributes may be associated with BRCA1's function in controlling the cell cycle and promoting arrest in response to DNA damage. To add to this, in human cancer cell lines, dysfunctional BRCA1 mutations led to increased expression of PLK‐1, resulting in cells lacking control over the spindle, which is essential for the separation of chromatids during both mitosis and meiosis (He et al. 2022). This can cause improper chromosomal segregation and aneuploidy, potentially leading to the formation of cancer. BRCA1 reduces the phosphorylation activity of PLK‐1, especially in the context of DNA damage. This prevents aberrant activity PLK‐1, thereby preventing the phosphorylation of proteins such as TP53, which in turn prevents p53‐induced apoptosis (Liu and Erikson 2003). Reduction of PLK‐1 activity by BRCA1 ensures sufficient response to DNA damage and prevents tumorigenesis. Nonetheless, greater understanding is required regarding whether there are other kinases controlled by BRCA1, and how BRCA1 modulates phosphorylation pathways through ATM.

## 4 BRCA1 Control on Acetylation Pathways

BRCA1 and ER⍺ interactions have consistently been an ambiguous topic, and their relationship is not fully understood. While a substantial body of work shows BRCA1 working in opposition to oestrogen signalling, BRCA1 also supports these pathways. For example, BRCA1 is known to cause the ubiquitination and degradation of ER⍺ (Eakin et al. 2007), but acts as a transactivator of the ER⍺ gene (Archey and Arrick 2017). Although the mechanism of this interaction is unclear, BRCA1 has been shown to regulate ERα through acetylation (Ma et al. 2010). Depletion of BRCA1 by RNAi increases acetylation of ER⍺, and overexpression of BRCA1 reduces acetylated ER⍺ (Ma et al. 2010). BRCA1‐BARD1 do not have any reported enzymatic acetylation activity, although this interaction is largely unknown. Acetylation of ERα is associated with increased DNA binding at ER elements, thereby increasing its role as a transactivator (Kim et al. 2006). When BRCA1 reduces ERα acetylation, it mitigates the effects of ERα‐associated functions such as cell proliferation (Miziak et al. 2023). Additionally, BRCA1 suppresses the expression of p300, a protein responsible for ERα acetylation, further regulating ERα (Kim et al. 2006; Ma et al. 2010).

Acetylation is essential for p53 as it increases the stability of the protein and its affinity to promoters in DNA (Tang et al. 2008). BRCA1 and ATM work in conjunction using p300 to acetylate p53, where BRCA1 phosphorylation is a prerequisite for forming a complex with p300 and p53, thereby enabling acetylation of p53 (Li et al. 2019). While the mechanics of this interaction remain unclear, phosphorylation of BRCA1 at the previously mentioned sites is indispensable, as when PP2Cδ inhibits phosphorylation of BRCA1 through the mitigation of ATM activity, this interaction abolishes p53 acetylation through BRCA1. This study postulates that this phosphorylation causes a conformational change, enabling the physical interaction between p300 and p53, thereby facilitating the formation of the complex. Acetylated p53 has greater stability and allows for more robust binding with low‐affinity promoters (Sakaguchi et al. 1998; Luo et al. 2004; Knights et al. 2006; Yamaguchi et al. 2009; Reed and Quelle 2014). Acetylation is a pivotal modification to p53, without which, key tasks such as apoptosis and arrest of the cell cycle are not possible (Tang et al. 2008). Triple‐negative breast cancers that are deficient in BRCA1 are suppressed through the restoration of p53 acetylation (Cao et al. 2021), further linking p53 acetylation and BRCA1 function. As such, BRCA1's coordination of p53 acetylation has significant consequences for suppressing tumorigenesis.

Vitamin D has an established role in anti‐tumorigenesis, where its metabolic products can control cell differentiation (Bustamante‐Madrid et al. 2024). Pickholtz et al. (2014) found that BRCA1 acts as a coactivator of the vitamin D receptor and interacts with the vitamin D‐responsive elements in the promoter for p21waf1, which is involved in the disruption of cell cycle progression as a response to DNA damage (Vigneron et al. 2006). This assembly at the promoter causes the acetylation of H3 and H4 histones, thereby increasing transcriptional availability and enhancing p21waf1 expression (Pickholtz et al. 2014). The absence of BRCA1 reduces acetylation at VDREs, specifically those located farther from the transcription start site, thereby reducing the anti‐tumorigenic activity of vitamin D and its analogues (Pickholtz et al. 2014). This suggests that BRCA1 is a co‐activator of VDR, indirectly controlling acetylation by regulating other enzymes that alter the chromatin landscape through its E3 ubiquitin ligase activity. However, a mechanistic understanding of how BRCA1‐BARD1 are precisely involved remains unknown. Vitamin D derivatives have anti‐tumorigenic properties (Dallavalasa et al. 2024). This co‐activator role of BRCA1 is in line with its canonical functions, through increasing transcriptional availability through acetylation.

## 5 BRCA1 Regulates Methylation

A common PTM that occurs on DNA, histones, and other proteins is methylation, which results in gene repression, where the DNA architecture changes, causing the DNA to pack tightly, thereby preventing access of transcription factors (Grau et al. 2023). BRCA1 associates with HP1 in an ATM‐dependent manner, resulting in methylation of chromatin at retrotransposons (Filipponi et al. 2013). HP1 proteins have the function of stabilising heterochromatin (Seman et al. 2023), and this complex is often observed near retrotransposon elements, such as L1 LINEs (Filipponi et al. 2013). L1 LINES are one of the most active retrotransposons in mammals (Brouha et al. 2003). These active retrotransposons can replicate by themselves using reverse transcriptase enzymes encoded in the L1 LINE (Schwertz et al. 2018) and move within the genome. The resulting genomic disruptions could lead to cancer. As a result, they require repression to maintain genomic stability, which is achieved through BRCA1‐induced methylation of these regions (Filipponi et al. 2013; Mita et al. 2020). BRCA1 is involved in the activation of DNMTs, methyltransferases that catalyse methylation of chromatin and other proteins. Their recruitment has also been shown to occur through HP1 (Smallwood et al. 2007; Filipponi et al. 2013). Although there have been no recent advances regarding the interaction between BRCA, HP1 and methylation, this complex allows BRCA1‐BARD1 to maintain at DNA damage sites by interacting with H3K9me2 (Wu et al. 2015). The role in global methylation is further established by the fact that BRCA1 is a transactivator for DNMT1 (Shukla et al. 2010). In the absence of BRCA1, global hypomethylation is observed due to the lack of DNMT1 to drive methylation, where repressed genes are expressed due to hypomethylation. This included proto‐oncogenes observed at higher levels in BRCA1‐deficient cells (Shukla et al. 2010). Hypomethylation of DNA is associated with genomic instability (Sheaffer et al. 2016) and prevents the activation of major cell cycle checkpoints (Barra et al. 2012). BRCA1's influence on global methylation directly contributes to its canonical roles of preventing tumorigenesis through maintaining genomic stability and effectively triggering cell cycle checkpoints.

BRCA1 is required for Meiotic Sex Chromosome Inactivation (MSCI), silencing of the X and Y chromosomes during spermatogenesis to prevent infertility (Royo et al. 2010). Current understanding suggests that BRCA1 contributes to changes to the heterochromatin architecture, which is essential for the development of the XY body and for successful MSCI (Broering et al. 2014). In mouse models, it is believed that FANCB, the BRCA1 interactor and DNA damage repair protein, is required for chromosomal methylation in MSCI (Kato et al. 2015). It remains unclear whether BRCA1 is necessary for FANCB to perform this role. Further research must be conducted to observe if abolishing BRCA1 function would interrupt FANCB methylation of sex chromosomes. However, it is suggested that, in this role, BRCA1 mediates chromatin modification of the sex chromosomes through methylation, phosphorylation, and ubiquitination (Abe et al. 2022). BRCA1 is not responsible for MSCI in all models. In *Caenorhabditis elegans*, the absence of the BRCA1‐BARD1 orthologues, BRC1‐BRD1, does not have any changes to methylation levels on the X chromosome, indicating that another protein(s) is responsible (Li et al. 2020). Furthermore, observations in *C. elegans* have shown that depletion of BRC‐1 and BRD‐1 does not influence methylation of H3K9me2 on the X chromosome (Li et al. 2020). Although BRC‐1 and BRD‐1 are not essential for this process, synergistic lethality was observed when MET‐2, a histone methyltransferase, and the BRC‐1‐BRD‐1 complex are simultaneously knocked down (Padeken et al. 2019). MET‐2, a methyltransferase responsible for H3K9me2, is required to repress satellite DNA, a function shared by BRCA1 (Padeken et al. 2019). This suggests that removing MET‐2 and the BRC‐1‐BRD‐1 complex leads to the expression of satellite DNA, resulting in DNA damage that is inadequately repaired in the absence of BRC‐1, ultimately causing cell death.

BRCA1‐like cancers refer to cancers that express phenotypes of a dysfunctional BRCA1 protein, but do not necessarily contain BRCA1 mutations. These include cancers with the BRCA1 promoter heavily methylated or other forms of aberrant transcriptional repression. Bioinformatic comparison of miRNA levels in BRCA1‐like cancers with those in non‐BRCA1‐like cancers found downregulation of the miR‐29 family in BRCA1‐like tumours (Santarosa et al. 2023). Other studies echoed this sentiment, finding that BRCA1 mutations resulted in a diminishing of miR‐29b‐1‐5p levels in mice (Milevskiy et al. 2018). miR‐29 miRNAs have been associated with reduced expression of methyltransferases such as DNMT3A and DNMT3B (Fabbri et al. 2007). Collectively, this suggests that dysfunctional BRCA1 downregulates miR‐29, leading to increased DNMT3A and DNMT3B mRNA levels, and therefore protein levels. This increase in DNMT3A and DNMT3B results in increased methylation of DNA, thereby restricting the transcription of genes (Santarosa et al. 2023). Therefore, an unstable genome has reduced access to DNA repair proteins and other mechanisms that maintain stability. Overexpression of miR‐155 in BRCA1‐like cancers compared to non‐BRCA1‐like cancers suggests that certain aspects of miRNA regulation were mediated through BRCA1 (Chang et al. 2011). Although BRCA1 may function as a transcriptional co‐regulator for these miRNAs, the findings from Santarosa et al. (2023) need to be verified through in vitro or in vivo studies that establish BRCA1's control over methylation through miRNA. Nonetheless, they do suggest that hypermethylation may occur in certain contexts of BRCA1 deficiency. BRCA1's regulation of the methylation landscape is essential for genomic stability. Hypermethylation of DNA is a feature typical of breast cancers, preventing the expression of tumour suppression genes (Li et al. 2006). Therefore, prevention of hypermethylation might be a function of BRCA1‐BARD1, impeding tumour development.

## 6 BRCA1 Negatively Regulates PARylation Through PARP1

PARylation is a PTM that controls protein recruitment and stability, and is associated with DNA damage response pathways (Masson et al. 1998). PARP‐1 is a DNA damage sensor that causes the addition of ADP‐ribose chains onto a molecule upon activation (Eustermann et al. 2011). Protein PARylation can alter transcriptional control and PARylation of histones leads to chromatin relaxation, facilitating transcription of DNA damage repair proteins (Tulin and Spradling 2003; Páhi et al. 2020). BRCA1 is regulated through PARylation, leading to significant clinical implications. Cells that have knockdown of BRCA1 and PARP‐1 experience synthetic lethality (Yang et al. 2025). Despite evidence that PARP1 regulates BRCA1, there is also evidence that BRCA1 regulates PARP1 (Hu et al. 2014; Lodovichi et al. 2023). Changes to BRCA1 concentrations alter levels of PARP1, where a reduction in BRCA1 leads to an increase in PARP1 levels and activity. Similarly, overexpressing BRCA1 results in a decrease in PARP1 (Li et al. 2014). This PARP1‐associated reduction in BRCA1 activity may stem from the availability of cellular NAD, a coenzyme used in PARylation. Less BRCA1 leads to an induction of PARP1 expression, and more NAD available for catalysis, resulting in an increase in PARP1 activity (Li et al. 2014). As NAD depletes, it further reduces BRCA1 expression, potentially leading to oxidative damage to DNA as ROS accumulate within cells. However, this regulatory interaction between BRCA1 and PARP1 has been primarily observed in human breast‐derived cell lines, and its conservation across model organisms remains unclear. A greater understanding is needed to determine whether BRCA1's influence on PARP1 expression and PARylation is context‐specific or part of a broader, conserved DNA repair mechanism. This regulatory interaction between BRCA1 and PARP1 could be a mechanism to prevent hyper‐PARylation, as Hyper‐PARylation depletes NAD^+^ and exhaustion of NAD^+^ initiates cell death (Hurtado‐Bagès et al. 2020; Santofimia‐Castaño et al. 2022). As such, BRCA1 regulation of PARP1 could prevent excessive cell death; however, this requires further understanding.

## 7 Gaps and Future Directions

Despite influencing proteins using different modifications, it is still not understood whether this functionality arises from BRCA1's E3 ubiquitin ligase capacity. Further experimentation is to be conducted using BRCA1 mutations, where E3 ligase capacity is dead, to identify if BRCA1 is associated with other downstream proteins that cause the addition of PTMs. In many of the interactions discussed in this review, the exact mechanism by which the BRCA1‐BARD1 complex acts remains unclear. Many of the findings do not identify whether BRCA1‐BARD1 plays the role of a coactivator, ligand, or transcriptional activator. Therefore, these associations could be due to downstream interactions, rather than direct physical interactions. Many BRCA1‐interacting proteins also remain to be identified. Due to this, the consequences of deletion or depletion of BRCA1 have yet to be mechanistically understood. This review has provided a perspective into the current understanding of BRCA1‐associated PTMs. Although some are gaining clarity, other PTMs, such as SUMOylation, are yet to be linked to BRCA1.

Hypermethylation and hypomethylation have devastating consequences, leading to cancer, and the regulation of these processes is important in preventing genomic instability (Li et al. 2006; Sheaffer et al. 2016). BRCA1 is linked to both the suppression and promotion of global methylation, suggesting a varied role regarding this PTM. Ubiquitination is the most understood function of the BRCA1‐BARD1 complex and coordinates HR to repair DNA, preventing mutations that lead to tumour formation. Regarding phosphorylation, BRCA1 regulates the activity of major kinases, including ATM, PLK‐1, AURKA, and AURKB (Moiola et al. 2012; Wang et al. 2014; Chabalier‐Taste et al. 2016). Interestingly, BRCA1 and ATM are observed to mutually regulate each other, where ATM phosphorylation of BRCA1 is important for ATM autophosphorylation and the phosphorylation of other ATM substrates (Moiola et al. 2012). As a major DNA damage sensor, this kinase activity is imperative for HR and cell cycle control (Bakr et al. 2015). If these pathways are not effectively activated, defects can lead to mutations, and precancerous cells can survive without triggering apoptosis or senescence. This further illustrates a conflicting role of BRCA1 regarding PTM function.

Understanding the direct role of BRCA1‐associated PTMs is further complicated due to the conflicting roles of BRCA1 in acetylation and PARylation. For example, BRCA1 expression is inversely related to ER⍺ acetylation (Ma et al. 2010), and BRCA1 has been implicated in the degradation of ER⍺, where overexpression and activation are highly associated with cell proliferation and cancer (Miziak et al. 2023). Conversely, BRCA1 is responsible for acetylating p53, increasing its stability (Li et al. 2019). Acetylated p53 has a greater ability as a transcription factor and can activate apoptosis and cell cycle checkpoints (Tang et al. 2008). When BRCA1 function is impaired, acetylation of p53 decreases, restoration of which recovers antitumorigenic activity in triple‐negative breast cancer (Cao et al. 2021). BRCA1 activation also restricts hyper‐PARylation by limiting PARP1 expression and activity, while hyper‐PARylation leads to cell death (Li et al. 2014). The clinical significance of BRCA1 in some of these PTMs has not yet been established, but further research could link these modifications to disease markers and other pathological pathways. Furthermore, elucidating the biochemical roles of the BRCA1‐BARD1 complex would unveil additional insights into carcinogenesis. Additionally, understanding how BRCA1‐BARD1 is involved in these intricate networks of PTMs would clarify the influence this complex has in maintaining cellular homoeostasis.

## Author Contributions

Nethma Hewa Waduge contributed to design, drafting and editing. Junhua Xiao contributed to editing. Gregory M. Davis contributed to design, drafting and editing.

## Funding

The authors have nothing to report.

## Conflicts of Interest

The authors declare no conflicts of interest.

## Data Availability

Data sharing not applicable to this article as no datasets were generated or analysed during the current study. No data was generated for the construction of this manuscript.
